# Variation in Serotonin Transporter Expression Modulates Fear-Evoked Hemodynamic Responses and Theta-Frequency Neuronal Oscillations in the Amygdala

**DOI:** 10.1016/j.biopsych.2013.09.003

**Published:** 2014-06-01

**Authors:** Christopher Barkus, Samantha J. Line, Anna Huber, Liliana Capitao, Joao Lima, Katie Jennings, John Lowry, Trevor Sharp, David M. Bannerman, Stephen B. McHugh

**Affiliations:** a Department of Experimental Psychology, University of Oxford, Oxford, United Kingdom; b Department of Physiology, Anatomy, and Genetics, University of Oxford, Oxford, United Kingdom; c Department of Chemistry, National University of Ireland, Maynooth, Ireland; d Department of Pharmacology, University of Oxford, Oxford, United Kingdom

**Keywords:** serotonin transporter, fMRI, amygdala, fear, tissue oxygen, theta oscillations

## Abstract

**Background:**

Gene association studies detect an influence of natural variation in the 5-hydroxytryptamine transporter (5-HTT) gene on multiple aspects of individuality in brain function, ranging from personality traits through to susceptibility to psychiatric disorders such as anxiety and depression. The neural substrates of these associations are unknown. Human neuroimaging studies suggest modulation of the amygdala by 5-HTT variation, but this hypothesis is controversial and unresolved, and difficult to investigate further in humans.

**Methods:**

We used a mouse model in which the 5-HTT is overexpressed throughout the brain and recorded hemodynamic responses (using a novel in vivo voltammetric monitoring method, analogous to blood oxygen level–dependent functional magnetic resonance imaging) and local field potentials during Pavlovian fear conditioning.

**Results:**

Increased 5-HTT expression impaired, but did not prevent, fear learning and significantly reduced amygdala hemodynamic responses to aversive cues. Increased 5-HTT expression was also associated with reduced theta oscillations, which were a feature of aversive cue presentation in controls. Moreover, in control mice, but not those with high 5-HTT expression, there was a strong correlation between theta power and the amplitude of the hemodynamic response.

**Conclusions:**

Direct experimental manipulation of 5-HTT expression levels throughout the brain markedly altered fear learning, amygdala hemodynamic responses, and neuronal oscillations.

The serotonin (5-hydroxytryptamine; 5-HT) transporter (5-HTT) is a key determinant of brain 5-HT function as it controls 5-HT availability at the synapse. There is a large natural variation in 5-HTT expression in the human population, approximately threefold between individuals (1). Current thinking is that this variation, in large part driven by the 16 or more 5-HTT gene polymorphisms discovered to date, is the source of large individual differences in personality, behavior, and brain disorder susceptibility. In this regard, gene association studies have reported that a common insertion/deletion polymorphism (producing long (l) and short (s) variants, respectively) in the 5-HTT gene upstream promoter region (5-HTTLPR) generates high (l/l) and low (s/s) expressing variants, with the s/s genotype conferring increased risk for anxiety-related traits (2) and affective disorders, especially when combined with environmental factors 3, 4, and the l/l genotype conferring reduced risk. As is common with gene-association studies, these findings are confounded by failed replications 5, 6 and need to be underpinned by a convincing neural substrate.

An attractive theory is that 5-HTT variation has an impact on emotionality and affective disorder susceptibility through modulation of the amygdala. This idea derives largely from human functional magnetic resonance imaging (fMRI) studies, which detect lower amygdala blood oxygen level–dependent (BOLD) responses to aversive cues in l/l versus s carriers 7, 8. However, a recent meta-analysis of available published and unpublished data sets found that the association between 5-HTT variation and amygdala reactivity was of borderline statistical significance (with no significant genotypic difference in 21 of 34 samples) and resolvable only through further large-scale, and thus impractical, imaging studies to control for study design and subject heterogeneity (9).

At the heart of the 5-HTTLPR debate lie two fundamental and as yet unanswered questions: 1) Does variation in 5-HTT expression influence how aversive cues are processed, and, if so, 2) what are the underlying neuronal mechanisms? Here, as an alternative to gene-association studies, we used a mouse model of genetically altered 5-HTT expression to address these questions. 5-HTT overexpressing mice (5-HTTOE) have two- to threefold greater 5-HTT expression than wild-type (WT) mice (10), mirroring the natural variation in humans 1, 11, 12, with 5-HTTOE mice approximating the human l/l genotype (10). Here we investigated aversive learning and amygdala activity in 5-HTTOE and WT mice during Pavlovian fear conditioning.

We recorded amygdala activity in two ways. First, to allow direct comparison with human neuroimaging, we recorded amygdala hemodynamic responses in behaving 5-HTTOE and WT mice. BOLD fMRI cannot be performed in freely moving rodents but recent advances in tissue oxygen (T_O2_) voltammetry offer equivalent hemodynamic measurements. T_O2_ signals are driven by the same neurovascular mechanisms as the BOLD signal and therefore provide a close hemodynamic surrogate, via intracerebrally implanted carbon paste microelectrodes 13, 14, 15, 16. Recently, we have shown that T_O2_ signals in the amygdala display a brain-region-specific discrimination between aversive and neutral cues during fear conditioning in rats (17). Simultaneously in the same mice, we measured neuronal activity in the form of local field potentials (LFPs).

Here we show that increased 5-HTT expression impairs fear learning and reduces amygdala hemodynamic responses and theta oscillations evoked by aversive cues. Finally, we show that the hemodynamic response amplitude is strongly correlated with theta oscillatory power in WT mice, but not in 5-HTTOE mice, suggesting a plausible neural basis for the 5-HTTLPR-related differences in human BOLD signals.

## Methods and Materials

For a full description of the methods, see Supplement 1.

### Subjects

Male 5-HTTOE and WT mice were generated on a CBA × C57BL/6J background, as described previously (10). Mice were approximately 5 months old at the time of surgery. The experiments were conducted in accordance with the United Kingdom Animals Scientific Procedures Act (1986) under project license 30/2561.

### Surgery

Mice were surgically implanted with a carbon paste electrode (CPE, 200-μm diameter) into the basolateral amygdala to measure T_O2_ and a silver electrode (125-μm diameter) into the basolateral amygdala of the contralateral hemisphere to measure LFPs, as described previously 14, 17. Right/left electrode positions for T_O2_/LFP recordings were counterbalanced across mice. Coordinates were −1.35 mm anterior/posterior, ±3.10 mm medial/lateral, and −5.00 mm dorsal/ventral, relative to bregma. Auxiliary and reference electrodes (200-µm diameter silver wire) were implanted into parietal cortex. A pedestal plug (MS363, Plastics One, Roanoke, Virginia) was secured with dental cement and skull screws. Mice were allowed to recover for at least 7 days after surgery.

### T_O2_ Measurements

T_O2_ signals were measured using constant potential amperometry, as described previously 14, 17, 18. When a constant potential (−650 mV relative to reference) is applied to an electrode implanted into the brain, O_2_ is electrochemically reduced on the electrode’s surface, inducing a current directly proportional to the local O_2_ concentration (19). Like the fMRI-BOLD signal, the T_O2_ signal is determined primarily by changes in local cerebral blood flow 16, 20.

### Fear Conditioning Procedures

Two different fear conditioning paradigms were used. A separate cohort of unoperated mice (*n* = 11 per genotype) were tested on a standard rodent fear conditioning paradigm to see if 5-HTT overexpression affected fear learning. Mice received two training trials (30-second tone followed by .3 mA, .5-second shock) in one context followed 24 hours later by two tone-alone presentations in a novel context.

The operated mice (*n* = 42; 22 WT, 20 5-HTTOE) were tested on a discriminative fear-conditioning paradigm. This behavioral paradigm differs from the standard fear-conditioning paradigm described above in that mice must learn to discriminate between two distinct auditory cues (tone and white noise), with one cue paired with footshock (conditioned stimulus; CS+) and the other cue never paired with footshock (CS–). This discriminative approach is commonplace in human fMRI 21, 22 and rodent electrophysiologic studies of fear 23, 24. Because any stimulus could potentially evoke amygdala activity, the CS– provides the necessary nonaversive control stimulus with which to compare CS+ evoked responses, akin to a subtraction task in fMRI.

Discriminative fear conditioning was performed over 5 consecutive days. On Day 1 (pre-exposure), mice were presented with the auditory cues (five 2900-Hz tones and five white noise stimuli, both 30 seconds in duration, presented in pseudorandom order), with no shocks administered. On Days 2 through 4 (training), the mice were placed into a different context and presented with the same auditory cues, but now one cue (tone or white noise, counterbalanced across mice) was always paired with coterminating footshock (.3 mA, .5 seconds), whereas the other cue was not. On Day 5 (fear memory recall), mice were placed into a novel context and presented with the auditory cues with no shocks administered. During all days, cue-evoked freezing behavior and amygdala T_O2_ responses and LFPs were recorded simultaneously in the same mice.

### Data Analysis

Behavior was recorded with a video camera and freezing was measured using Videotrack (Viewpoint, Champagne Au Mont D’Or, France) or NIH Image (25). A freezing “difference score” was calculated as follows: percent freezing during the 30-second cue presentation minus percent freezing during the 30 seconds before cue presentation (i.e., positive freezing scores indicate increased freezing to the cue and negative freezing scores indicate decreased freezing to the cue relative to the precue period).

Cue-evoked T_O2_ responses were calculated by subtracting the mean T_O2_ signal in the 5 seconds before CS onset (i.e., baseline) from the T_O2_ signal during the 30-second CS presentation. This yielded a 30-second ΔT_O2_ signal, which was then divided into fifteen 2-second timebins (i.e., 0–2, 2–4, 4–6 . . . 28–30 seconds), with each data point equal to the mean value during each 2-second timebin (Figure S2 in Supplement 1) (17).

LFPs were band-pass filtered between 1 and 45 Hz. Power spectra were calculated using a fast Fourier transform over the first 10 seconds of CS presentation and were averaged over the five CS+ versus the five CS– trials on each day for each mouse. To compare across mice, spectra were normalized by expressing the power in each frequency bin as a proportion of the total power between 1 and 45 Hz (Figure S2 in Supplement 1).

### Histology

Electrode placements were determined at the end of the experiment. Mice were transcardially perfused with physiologic saline (.9% NaCl), followed by 10% formol saline (10% formalin in .9% NaCl). Coronal sections (40 μm) were cut on a freezing microtome and stained with cresyl violet. Only mice with confirmed electrode placements in the basolateral amygdala were used in the T_O2_ and LFP analyses (Figure S1 in Supplement 1).

Serotonin transporter binding of [^3^H]citalopram in the amygdala was assessed using autoradiography in naive mice (5-HTTOE: *n* = 6; WT: *n* = 5; aged 3–6 months). High-performance liquid chromatography with electrochemical detection was used to measure amygdala tissue levels of 5-HT in a separate cohort of mice (*n* = 5 per group) (26).

### Statistical Procedures

Data were analyzed using *t* tests, analysis of variance (ANOVA), or Pearson correlation. The familywise error was set at α = .05. Unless otherwise stated, all graphs show the mean ± 1 standard error of the mean (SEM).

## Results

### Amygdala 5-HTT Expression Is Higher in 5-HTTOE Than WT Mice

First, we established that amygdala 5-HTT expression, measured by [^3^H]citalopram binding, was 2.7-fold higher in 5-HTTOE compared with WT mice (121.4 ± 4.3 vs. 44.4 ± 5.5 fmol/mg; *t*_9_ = 11.2, *p* < .001). Moreover, high-performance liquid chromatography revealed lower amygdala 5-HT tissue levels in 5-HTTOE compared with WT mice (4.4 ± .9 versus 7.6 ± 1.0 pmol/sample; *t*_8_ = 2.5, *p* < .05; Table S1 in Supplement 1) 10, 27. Thus the genetic modification led to higher 5-HTT expression in the amygdala and this, in turn, resulted in lower 5-HT tissue levels.

### 5-HTT Overexpression Impairs Conditioned Fear

**Figure 1:**
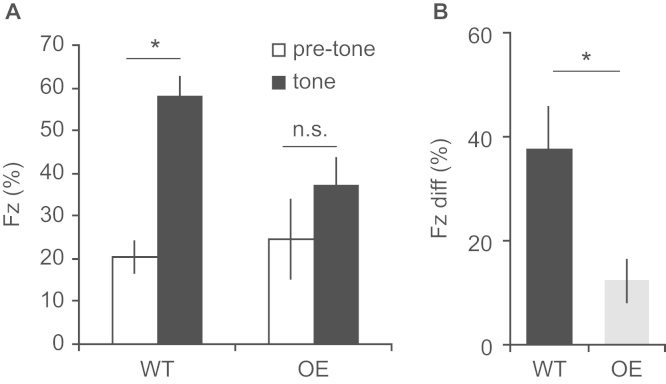
Standard fear conditioning experiment. **(A)** Wild-type (WT) mice exhibit significantly increased freezing (Fz) to a conditioned auditory tone (compared with the pretone period) during fear memory recall, whereas this did not reach significance for 5-hydroxytryptamine transporter overexpressing (OE) mice (genotype × phase interaction: *F*_1,20_ = 8.3, *p* = .009; pretone versus tone, WT: *p* < .001; OE: *p* = .07). **(B)** When pretone Fz was subtracted from the tone-evoked freezing, WTs exhibited significantly higher Fz than 5-HTTOE mice. *p ≤ .01. Fz diff, freezing difference score.

Next, we investigated whether variation in 5-HTT expression affected aversive learning, using a standard rodent fear-conditioning paradigm. During training, unconditioned responses to the tone and shock did not differ between WT and 5-HTTOE mice (all *F*s < 1, *p* > .5). In addition, WT and 5-HTTOE mice showed indistinguishable acoustic startle responses (see Supplement 1). These results suggest normal ability to hear the tone and normal shock responsivity in 5-HTTOE mice. However, during the fear memory recall test, WT mice froze significantly more during the conditioned tone than the pretone period, whereas this did not reach significance in 5-HTTOE mice (genotype × phase interaction: *F*_1,20_ = 8.3, *p* = .009; tone versus pretone, WT: *p* < .001; 5-HTTOE: *p* = .07; Figure 1A). When a difference score was calculated (tone freezing – pretone freezing), WTs froze significantly more than 5-HTTOE mice (*t*_20_ = 2.9; *p* = .01; Figure 1B), thereby demonstrating impaired fear learning in 5-HTTOE mice.

### 5-HTT Overexpression Reduces Amygdala Hemodynamic Responses to Aversive Cues

Next, to determine if variation in 5-HTT expression affected amygdala activity in mice as has been suggested by human neuroimaging studies, we investigated T_O2_ responses and neuronal oscillations during discriminative fear conditioning. Before any cue-shock pairings, the mice were pre-exposed to the auditory cues. Importantly, there were no differences between WT and 5-HTTOE mice in their behavioral, hemodynamic or electrophysiological responses to the auditory cues before training (no effect of genotype or interactions involving genotype; Figure S3 Supplement 1).

**Figure 2:**
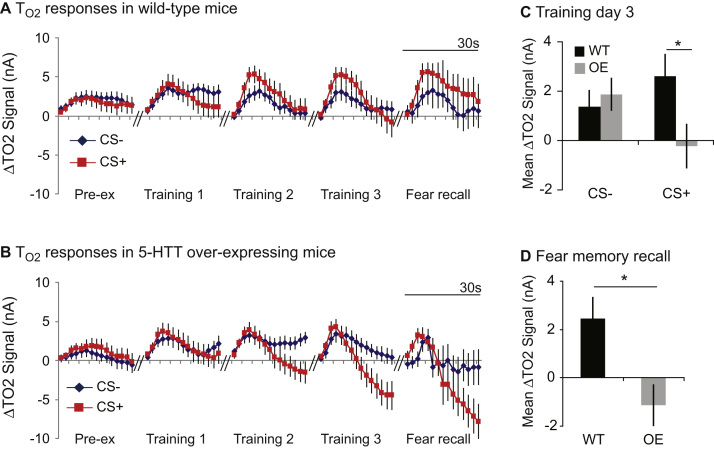
Cue-evoked tissue oxygen (T_O2_) signals in wild-type (WT) and 5-hydroxytryptamine transporter (5-HTT) overexpressing (OE) mice during discriminative fear conditioning for the pre-exposure (Pre-ex) day, the 3 days of training, and the first trial of fear memory recall. **(A)** In WT mice, conditioned stimulus (CS)+ evoked responses (red) increased relative to CS– evoked responses (blue) over training. **(B)** In 5-HTTOE mice, CS+ evoked responses were not higher than CS– evoked responses and had a pronounced “negative tail” by Training Day 3. **(C)** Mean CS+ (but not CS–) evoked T_O2_ responses were significantly higher in WTs than 5-HTTOEs by Training Day 3. **(D)** Mean T_O2_ responses were significantly higher in WTs than 5-HTTOEs during fear memory recall. *p < .05.

Fear conditioning led to marked changes in the amplitude and shape of the cue-evoked T_O2_ responses in the amygdala, with strikingly different responses in 5-HTTOE compared to WT mice. In WT mice, CS+ evoked T_O2_ responses increased in amplitude relative to CS– evoked responses over the course of training (Figure 2A). Specifically, the peak CS+ response (occurring approximately 8–10 seconds after CS+ onset) was higher than the peak CS– response. In contrast, in 5-HTTOE mice, peak CS+ and CS– evoked responses did not differ and, by the end of training, CS+ evoked T_O2_ responses were markedly lower than CS– evoked responses during the last approximately 10 to 15 seconds of CS presentation (Figure 2B). Importantly, CS+ evoked responses were higher in WT than 5-HTTOE mice.

Analysis (ANOVA: genotype_2_ × day_3_ × CS type_2_ × timebin_15_ × S_33_) confirmed that T_O2_ responses were significantly greater in WT than 5-HTTOE mice by Training Day 3 (genotype × CS type × day interaction: *F*_2,60_ = 3.9; *p* = .03; genotype × CS type interaction for day 3: *F*_1,31_ = 7.0; *p* = .01; Figure 2C). This was driven by genotypic differences in CS+ evoked T_O2_ responses (*p* < .05), with no genotypic differences in CS- evoked T_O2_ responses (*p* = .6; Figure 2C). Thus, fear-evoked T_O2_ responses in the amygdala were significantly higher in WT than 5-HTTOE mice during training.

A strong genotypic difference was also evident during fear memory recall. WT mice exhibited higher T_O2_ signals than 5-HTTOE mice (main effect of genotype: *F*_1,31_ = 8.9; *p* = .005), due to higher CS+ evoked responses (genotype × CS type × timebin interaction: *F*_14,434_ = 2.1; *p* = .01; with higher CS+ responses in WT than 5-HTTOE mice 10 to 30 seconds after cue onset, *p* < .05; Figure 2D). There were no genotypic differences in CS– evoked responses at any time point (Figure S4 in Supplement 1). Thus, 5-HTT overexpression resulted in lower amygdala hemodynamic responses specifically to aversive cues.

### 5-HTT Overexpression Impairs Discriminative Fear Learning but This Is Ameliorated with Additional Training

**Figure 3:**
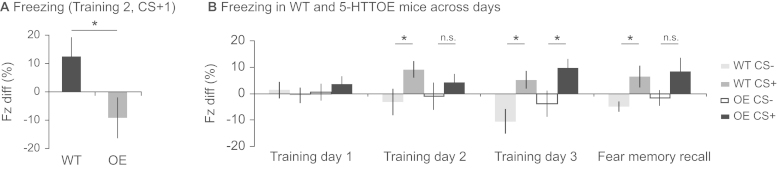
Cue-evoked freezing behavior in wild-type (WT) and 5-hydroxytryptamine transporter (5-HTT) overexpressing (OE) mice during discriminative fear conditioning. **(A)** WTs froze more than 5-HTTOE mice during the first conditioned stimulus (CS)+ presentation on Training Day 2. **(B)** WT mice discriminated between the CS+ and CS– on Training Days 2 and 3 and the fear memory recall day, but discrimination was seen in 5-HTTOE mice on Training Day 3 only, with a trend on the fear memory recall day. Each bar represents the mean freezing difference score (Fz diff) ± 1 SEM. *p < .05; **p = .07. n.s., not significant.

Behaviorally, 5-HTTOE mice exhibited impaired learning during discriminative fear conditioning, confirming the behavioral phenotype found with the standard fear-conditioning paradigm. On Training Day 2, WT mice froze significantly more than 5-HTTOE mice during the first CS+ presentation of the session (CS type × genotype interaction: *F*_1,40_ = 4.7, *p* = .04; CS+: WT > 5-HTTOE mice, *p* = .03; Figure 3A). Thus, the impairment in 5-HTTOE mice was seen at approximately the same time point during the standard and discriminative fear conditioning paradigms (i.e., after 1 day of training).

However, with additional training, 5-HTTOE mice learned to discriminate between the CS+ and CS–, and they were indistinguishable from WT mice in terms of freezing levels by Training Day 3 (Figure 3B). Analysis of freezing responses over all training trials (ANOVA: genotype_2_ × day_3_ × CS type_2_ × trial_5_ × S_42_) revealed that mice froze significantly more during CS+ than CS– trials overall (main effect of CS type: *F*_1,40_ = 6.3; *p* = .02) and showed greater discrimination later in training (CS type × day interaction: *F*_2,62_ = 6.8; *p* = .002). However, whereas WT mice showed significant discrimination between the CS+ and CS– by Training Day 2, 5-HTTOE mice only discriminated on Training Day 3. Thus, there was a behavioral impairment in the 5-HTTOE mice initially but their CS+/CS– discrimination was comparable to WT mice by Training Day 3. This was in marked contrast to the hemodynamic signals, which were still different between the two genotypes by the end of training.

### 5-HTT Overexpression Reduces Theta Oscillations to Aversive Cues

**Figure 4:**
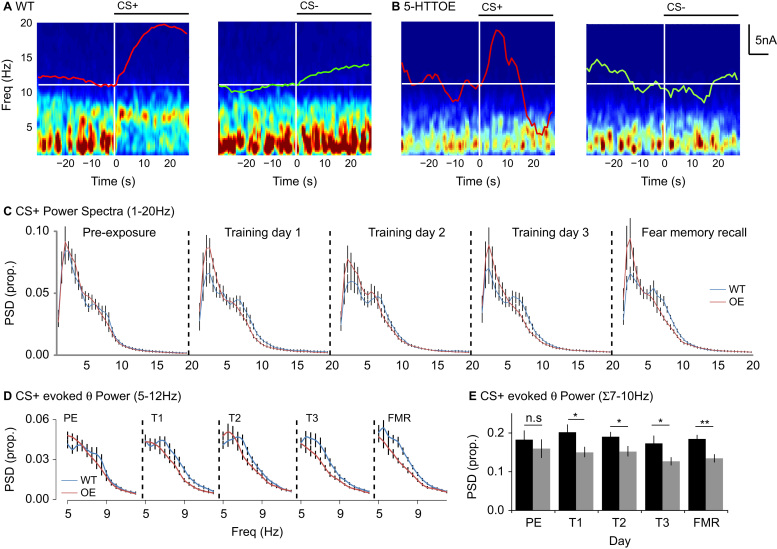
Cue-evoked neuronal oscillations in wild-type (WT) and 5-hydroxytryptamine transporter (5-HTT) overexpressing (OE) mice during discriminative fear conditioning. Representative spectrograms for a WT **(A)** and a 5-HTTOE **(B)** mouse on Training Day 3 showing the 30 seconds before and the 30 seconds during conditioned stimulus (CS) presentation (CS+: leftmost panel, CS– center left panel), with simultaneously recorded tissue oxygen (T_O2_) responses overlaid in red (CS+) or green (CS–). The CS+ evoked a shift from delta-dominant to theta-dominant oscillations in WT mice, which was attenuated in 5-HTTOE mice. **(C)** Power spectra (expressed as a proportion of the total spectral power between 1–45 Hz) for CS+ trials in WT (blue) and 5-HTTOE mice (red), showing lower delta activity (1–4 Hz) and higher theta activity (5–10 Hz) in WT mice. **(D)** CS+ evoked theta power was higher in WT than 5-HTTOE mice during training (T1–T3) and fear memory recall (FMR) day but not during pre-exposure (PE). **(E)** As in panel D but showing the summed theta power between 7 and 10 Hz. *p < .05; **p < .01. Freq, frequency; n.s., not significant; PSD (prop.), proportional power spectral density.

The different amygdala hemodynamic responses seen in WT versus 5-HTTOE mice were accompanied by genotypic differences in neuronal oscillations in the simultaneously recorded LFP. In WTs, aversive (CS+) but not neutral (CS–) cues evoked a shift from delta-dominant (1–4 Hz) to theta-dominant (5–10 Hz) neural oscillations (Figure 4A; Figure S5 in Supplement 1). This delta-to-theta shift was significantly reduced in 5-HTTOE mice, with markedly lower theta power between 7 and 10 Hz (see Figure 4B–D; Figure S6 in Supplement 1).

Analysis of theta oscillations over all days of training (ANOVA: genotype_2_ × day_5_ × CS type_2_ × S_25_) revealed significantly higher theta power in WT compared with 5-HTTOE mice (main effect of genotype: *F*_1,23_ = 5.2, *p* = .03. Analyses of individual days revealed that changes in theta power evoked by aversive cues (CS+) were significantly higher in WT than 5-HTTOE mice during the 3 training days and the fear memory recall test (all *F*s > 4.8, *p* < .04; Figure 4D,E), but there were no differences during the pre-exposure session (*F* < 1.1, *p* > .3). There were no significant genotypic differences in CS- evoked theta power on any day. Thus, 5-HTT overexpression reduced theta oscillations specifically to aversive cues.

### Theta Power Is Correlated with Amygdala T_O2_ Responses in WT Mice

**Figure 5:**
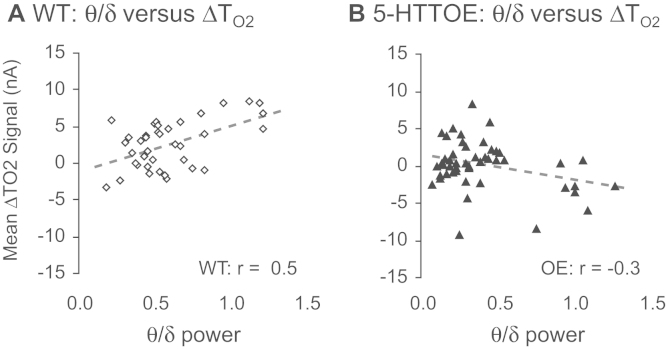
**(A)** For aversive cue (conditioned stimulus [CS]+) evoked responses, the ratio of theta-to-delta oscillatory power was significantly correlated with tissue oxygen (T_O2_) amplitude in wild-type (WT) mice. **(B)** This correlation was absent in 5-hydroxytryptamine transporter overexpressing (OE) mice. Note that each data point represents the mean theta:delta ratio versus mean T_O2_ response amplitude for each mouse on each of the 3 training days and the fear memory recall day.

Finally, to investigate the relationship between theta oscillations and T_O2_ responses, we plotted CS+ evoked changes in theta power (calculated by dividing the sum of theta power by the sum of delta power) against the mean T_O2_ response (i.e., the mean T_O2_ signal during each 30s CS+ presentation), collected over the 3 days of training and the fear memory recall test. The plots revealed that, in WT mice, the mean CS+ evoked T_O2_ amplitude was significantly correlated with CS+ evoked changes in theta power (*r* = .52, *n* = 40 observations; *t*_38_ = 3.8; *p* = .001, Figure 5A). This correlation was strikingly absent in 5-HTTOE mice (*r* = −.32, *n* = 48 observations; Figure 5B). This suggests that 1) the shift to theta-dominant oscillations evoked by aversive cues is a key mechanism underlying the amygdala hemodynamic response and 2) this delta-to-theta shift is acutely sensitive to variation in 5-HTT expression.

## Discussion

Here we demonstrate that direct, experimental manipulation of 5-HTT expression in mice, genetically engineered to model physiologically relevant increases in 5-HTT expression levels in humans, alters amygdala activity and amygdala-dependent behavior. Increased 5-HTT expression throughout the brain resulted in impaired fear learning, and aversive cues evoked significantly lower amygdala hemodynamic responses and theta oscillations in 5-HTTOE mice compared with WT controls. Moreover, theta oscillatory power and hemodynamic responses were highly correlated in WT mice but this correlation was absent in 5-HTTOE mice. Thus, modulation of theta oscillations provides a potential neuronal mechanism by which 5-HTT expression influences amygdala hemodynamic responses and fear learning.

### Increased 5-HTT Expression Is Associated with Reduced Amygdala Activity

Despite more than 30 human neuroimaging studies, the influence of the 5-HTTLPR genotype on amygdala BOLD responses remains equivocal. There may be many reasons why human neuroimaging studies have failed to resolve this issue (9). For example, 5-HTT expression levels are rarely measured in human studies, and polymorphic variation in the 5-HTTLPR is only one of several factors with the potential to influence 5-HTT expression (28). Indeed, although there is a reliable influence of the 5-HTTLPR on 5-HTT expression in vitro, several neuroimaging studies have failed to replicate this finding in vivo 29, 30. The strength of our approach is that we have directly manipulated and measured 5-HTT expression levels, which are two- to threefold higher in 5-HTTOE than WT mice (10). Therefore, our finding of significantly blunted fear-evoked amygdala T_O2_ responses in 5-HTTOE mice demonstrates unequivocally that 5-HTT expression influences amygdala hemodynamic responses.

### Increased 5-HTT Expression Is Associated with Reduced Theta Oscillations

Importantly, the current study found that 5-HTT expression influenced theta oscillations as well as hemodynamic responses. Specifically, 5-HTTOE mice exhibited reduced theta power evoked by fearful stimuli. Theta oscillations are consistently observed in the amygdala during fear conditioning 31, 32, 33 and can be intrinsically generated by amygdala neurons 32, 34. However, it is debated whether amygdala theta, as observed at a population level in the LFP, is locally generated or volume conducted from neighboring regions such as the hippocampus (see Supplement 1 for further discussion). Without concomitant single-unit recordings, caution is merited in attributing the source of these oscillations to the amygdala.

Nevertheless, current thinking is that theta oscillations may enhance plasticity locally or across a wider network of structures to facilitate the encoding and/or subsequent consolidation of fear memories 35, 36. Theta-burst stimulation patterns elicit synaptic plasticity in amygdala tissue slices and, importantly, simultaneous activation of 5-HT receptors during theta burst stimulation transforms short-term into long-term potentiation (37). Moreover, both footshock and aversive conditioned stimuli evoke 5-HT release in the amygdala 38, 39, 40, suggesting that 5-HT input may act to signal aversive events. Thus, during fear conditioning, theta oscillations, at least in part driven by 5-HT input, may facilitate the process whereby an initially neutral stimulus is transformed into an aversive conditioned stimulus, capable of evoking a powerful and long-lasting emotional response.

### Increased 5-HTT Expression is Associated with Reduced Fear Learning

Consistent with this, 5-HTTOE mice exhibited markedly impaired fear memory recall in the standard rodent fear conditioning paradigm and were also impaired in the discriminative paradigm after one day of training, although they learned the discrimination with continued training. This fear learning deficit mirrors the observation that human l/l homozygotes show lower skin conductance responses than s carriers following the pairing of simple visual stimuli with electric shock 41, 42. It is also consistent with the reduced amygdala activation seen in the present study. The importance of the amygdala for fear learning is well established in both animal lesion and human imaging studies 43, 44, 45, although the amygdala operates in concert with several other brain areas to mediate fear behavior. Nevertheless, deficits in fear learning in 5-HTTOE mice are consistent with the reduced amygdala activity found in these animals.

However, increased 5-HTT expression did not completely block discriminative fear conditioning but reduced the rate of learning. Notably, there were no behavioral differences between the groups by the end of training. Importantly, at this stage, there were still robust differences in the amygdala hemodynamic responses and theta oscillations between the two genotypes. At one level this is important because it means that the genotypic differences in amygdala activity cannot be explained by differences in the behavior of the two groups at the time of recording. Nevertheless, there is a disconnection between amygdala hemodynamic responses and freezing behavior. However, this result accords with other rodent studies showing that amygdala neuronal activity does not provide a simple readout of freezing behavior 23, 24. Moreover, the 5-HTTLPR-related differences in amygdala BOLD signals in humans are seen in the absence of any behavioral differences in the emotional face matching task or differences in anxiety-related traits 7, 8, 46.

However, note also that amygdala T_O2_ responses in 5-HTTOE mice clearly discriminated between the aversive (CS+) and nonaversive (CS–) cues by the end of training, except in the opposite direction to WTs. 5-HTTOE mice exhibited markedly lower signals during the last 10 to 15 seconds of the CS+ cue compared with both the pre-CS+ baseline and CS– trials (see Figure 2B). This is a surprising, but robust, result. This is unlikely to be a “ceiling effect” or an artifact of the difference measure used (i.e., the ΔT_O2_ change from baseline) because the decrease below baseline did not occur on CS- trials. Moreover, it emerged over the course of fear learning and was most evident on Training Day 3, when the 5-HTTOE mice also showed successful behavioral discrimination between the CS+ and CS–, comparable to WT mice. Thus, amygdala T_O2_ responses in 5-HTTOE mice did discriminate between the cues but in a qualitatively different way from WT mice.

### Theta Oscillations Are Correlated with Amygdala T_O2_ Responses

This is the first study to show a relationship between theta oscillations and the amplitude of the amygdala hemodynamic response. Aversive cue-evoked changes in theta power were significantly correlated with T_O2_ amplitude in WT but not 5-HTTOE mice. In humans, aversive stimuli evoke increased theta oscillations in the amygdala, as measured by magnetoencephalography (47), as well as amygdala BOLD signals (8), which strengthens the link between theta oscillations and hemodynamic responses. However, the lack of correlation between the theta:delta ratio and the amygdala T_O2_ response amplitude in 5-HTTOE mice could reflect the possibility that hemodynamic measures are no longer accurately reporting the underlying neuronal activity in 5-HTTOE mice. In other words, in addition to affecting hemodynamic responses and theta oscillations, 5-HTT expression may influence the relationship between them. A detailed analysis of how 5-HTT expression influences neurovascular coupling is beyond the scope of this report but it merits further investigation. Moreover, in any case where neurovascular coupling is altered by a disease-relevant manipulation (such as a genetic polymorphism), this would represent a major challenge for interpreting human neuroimaging studies.

### Whole-Brain Versus Amygdala-Specific Effects

Our data demonstrate that variation in 5-HTT expression has a significant effect on amygdala activity and fear learning. However, because 5-HTT expression is upregulated throughout the brains of 5-HTTOE mice (10), the behavioral and physiologic phenotype we report could be due to altered 5-HTT expression in brain regions outside the amygdala. Although this is an important caveat in the interpretation of our data, these mice were specifically generated to model the global variation in 5-HTT expression seen in humans, which is not restricted to the amygdala. Nevertheless, in the present study, the consequences of this whole-brain genetic manipulation are clearly manifest in altered amygdala signals.

### Conclusions

To date, there is only equivocal evidence that 5-HTTLPR genotype affects amygdala hemodynamic responses in humans. Here we provide compelling evidence that whole-brain changes in 5-HTT expression influences amygdala activity and aversive cue processing in mice.
