# Supplementary material for: Variation in Serotonin Transporter Expression Modulates Fear-Evoked Hemodynamic Responses and Theta-Frequency Neuronal Oscillations in the Amygdala
Source: Biol Psychiatry. 2014 Jun 1;75(11):901–8. doi: 10.1016/j.biopsych.2013.09.003 (PMC4032572; doi:10.1016/j.biopsych.2013.09.003)
Supplement: Supplementary file 1 — Supplementary data [file mmc1.pdf]

Variation in Serotonin Transporter Expression Modulates Fear-Evoked Hemodynamic Responses and Theta-Frequency Neuronal Oscillations in the Amygdala

Supplemental Information

Supplementary Methods 2
Subjects 2
Surgery 2
Opposing-side Implantation of CPE and LFP Electrodes 3
Apparatus 3
Tissue O_2 Voltammetry 4
Standard Rodent Fear Conditioning Paradigm 5
Discriminative Fear Conditioning Paradigm 6
Acoustic Startle 7
Data Analysis 8
Behavior 8
Tissue oxygen signals 8
Local Field Potentials 9
Histology 10
Determination of Electrode Placements 10
Quantification of Serotonin Transporter Binding Levels 11
Neurochemical Analysis of 5-HT and 5-HIAA in Amygdala Tissue 11
Statistical Procedures 12
Supplementary Results 13
Acoustic Startle 13
Supplementary Discussion 14
Are Amygdala Theta Oscillations Locally Generated or Volume Conducted? 14
Figure S1. Histology 16
Figure S2. Methods for tissue oxygen and local field potential analyses 17
Figure S3. Discriminative fear conditioning: pre-exposure to the auditory cues 19
Figure S4. Aversive cue and non aversive cue evoked tissue oxygen signals in wild-type and 5-HTTOE mice during training and fear memory recall 21
Figure S5. Peri-stimulus responses to the CS+ on training day 3 in four different WT mice 22
Figure S6. Peri-stimulus responses to the CS+ on training day 3 in four different 5-HTTOE mice 24
Figure S7. Acoustic startle responses 26
Table S1. 5-HTT expression levels and 5-HT tissue levels in the amygdala of wild-type and 5-HTT over-expressing mice. 27
Supplemental References 28

Supplementary Methods

Subjects

Male serotonin transporter over-expressing (5-HTTOE) mice and their wild-type (WT) littermates were generated on a CBA × C57BL/6J background, as described previously (1), and bred at the University of Oxford. Mice were maintained on a 12/12 hr light/dark cycle (lights on 7 am; off 7 pm) with ad libitum food and water. Mice were housed in groups of 2-4 before surgery and individually after surgery. They were approximately 5 months old at the time of surgery. The experiments were conducted in accordance with the United Kingdom Animals Scientific Procedures Act (1986) under project license 30/2561 and approved by local ethical review.

Surgery

Mice were surgically implanted with one carbon paste electrode (CPE) into the basolateral amygdala (BLA) of one hemisphere (to measure tissue oxygen) and another electrode into the BLA of the contralateral hemisphere (to measure local field potentials (LFPs)) under isoflurane anesthesia. Approximately equal numbers of mice received left CPE / right LFP and right CPE / left LFP placements. CPEs were constructed from Teflon-coated 200 µm diameter silver wire (~270 µm coated diameter, Advent Research Materials, Oxon, UK) with the insulation pulled down the wire to produce a 2 mm cavity which was subsequently packed with carbon paste and smoothed (2). LFP electrodes were made from 125 µm diameter silver wire (~177 µm coated diameter, Advent). Coordinates for BLA implantations were -1.35 mm anterior/posterior, ±3.10 mm medial/lateral and -5.00 mm dorsal/ventral, relative to bregma. Auxiliary and reference electrodes (200 µm diameter silver wire) were implanted into parietal

cortex. Each electrode was soldered to a gold pin (E363/0, Plastics One, Roanoke, VA, USA), which was inserted into a pedestal plug (MS363, Plastics One) and secured with skull screws and dental cement ('Simplex Rapid', Associated Dental Products, Wilts, UK). Mice were allowed to recover for at least seven days after surgery.

Opposing-side Implantation of CPE and LFP Electrodes

We implanted LFP and T_{O2} electrodes into contralateral BLA sites, rather than into the same hemisphere, to minimize tissue damage to the BLA, which is a relatively small structure in a mouse (see Figure S1). It is possible, therefore, that differences in responses between left and right BLA could introduce variability when comparing neuronal oscillations with tissue oxygen (T_{O2}) responses. However, we do not think that this impacts on our results for three reasons. First, left/right placements were counterbalanced across mice so approximately equal numbers of T_{O2} and LFP electrodes were in a given hemisphere. Second, this counterbalancing was equivalent in WT and 5-HTTOE mice. Third, previous studies have shown that LFP responses (e.g. theta oscillations) show remarkably strong coherence across hemispheres (3).

Apparatus

T_{O2} signals were measured using constant potential amperometry, as described previously (4, 5). A constant potential (-650 mV relative to a reference electrode) was applied to the CPEs using a low-noise potentiostat ('Biostat,' ACM Instruments, Cumbria, UK). Mice were connected to the potentiostat via a 6-channel rotating commutator (SL6C, Plastics One) held on a counter-weighted arm (PHM-110P1, Med Associates) using screened cables (363-363 6TCM, Plastics One). A Powerlab® 8/30 (AD Instruments Ltd, Oxon, UK) was used for analogue /

digital conversion and data were collected on a Windows PC running Chart® v5 software (AD Instruments). LFPs were recorded using a differential amplifier (DP-301, Warner Instruments, CT, USA) or using the potentiostat with no potential applied, in which case the potentiostat acts as a differential amplifier. T_{O2} and LFPs were sampled continuously at 4 kHz.

Tissue O_2 Voltammetry

Applying a potential (-650 mV) to an electrode results in the electrochemical reduction of dissolved O_2 on the surface of the electrode, inducing an electrical current that is measured by the potentiostat. The availability of O_2 for this two-step reaction ($O_2 + 2H^+ + 2e^- \rightarrow H_2O_2$; $H_2O_2 + 2H^+ + 2e^- \rightarrow 2H_2O$) is determined by the local T_{O2} concentration. Thus, changes in O_2 concentration around the tip of the electrode produce directly proportional changes in the measured Faradaic current (6).

The area of sensitivity is estimated to be a sphere with diameter twice the electrode surface diameter, i.e. a 200 µm diameter electrode has ~400 µm diameter sphere of sensitivity (see also 7, 8). The spatial resolution of the T_{O2} electrode is sufficient to detect T_{O2} differences between lamina in rat whisker barrel cortex, i.e. approximately ~400 µm (9).

T_{O2} responses, like blood-oxygen-level dependent responses, are driven by changes in cerebral blood flow (CBF) and are dependent upon local neuronal activity (see (10), Figure 1). Infusion of the AMPAR antagonist CNQX or the $GABA_A$ agonist muscimol lead to reductions in both CBF and T_{O2} response amplitude (11, 12). Moreover, blocking sensory transduction in the peripheral nervous system (e.g. by applying the sodium channel blocker lidocaine to the whisker pad of rats) blocks both neuronal activity and T_{O2} responses in the somatosensory cortex (9).

Standard Rodent Fear Conditioning Paradigm

Fear conditioning in unoperated mice took place in an operant chamber (17 cm long × 11.5 cm wide × 20 cm high) located in a sound-insulated box. The walls and lid of the chamber (illuminated by a ceiling mounted light) were composed of clear Perspex whilst the floor of the chamber consisted of 19 stainless steel bars approximately 8.5 mm apart, through which scrambled shocks were delivered (0.3 mA, 0.5 s duration, San Diego Instruments shock generator). During training a plastic cube scented with artificial "apple pie" odor was placed next to the conditioning chamber inside the sound-insulated box to give it a distinct odor from the testing context. Between each trial all faeces/urine were removed and the boxes were cleaned.

Black-and-white video images of the mice were captured by a wide-angle video camera attached to the ceiling of the chamber, and relayed to a computer via a Panasonic video recorder (NVSD400). Video data were analysed using a Videotrack (vNT4.0) automated tracking system (Viewpoint, Champagne Au Mont D'Or, France) with a low and high activity threshold settings. "Freezing" was defined as periods during which movement fell below the lower activity threshold. With this threshold breathing movements did not register as activity (i.e. absence of freezing), but small purposeful movements (e.g. sniffing) were detected as activity. The amount of time spent freezing per 30 s time bin was calculated. For measurement of the unconditioned response to the tone and the unconditioned burst activity response to the shock, time bins of 1 s were used.

The training session began with a 6 min acclimatization period, during which low-volume white noise occurred. This was followed by a 30 s auditory tone. On tone offset mice received a 0.5 s footshock (0.3 mA). After a further 3 min, a second tone/shock pairing was delivered, followed by a further 3 min period before the mice were removed. Twenty four hours

after the training session, fear memory recall for the cue was tested in a novel environmental context (a round plastic chamber with patterned walls, smooth floor, and a distinctive "chicken" odor). Mice experienced two 30 s presentations of the tone (without footshock) during the 5 min session.

Discriminative Fear Conditioning Paradigm

Discriminative fear conditioning in mice with recording electrodes was conducted in one of three operant chambers (ENV-307A, Med Associates Inc., Lafayette, IN, USA), each with distinct visual and olfactory cues to aid context discrimination. Stimulus delivery was controlled by a custom-written script in the MED-PC language. Timed TTL-pulses to the AD converter ensured that stimulus delivery was synchronized with the electrophysiological recordings at 1 ms resolution.

Discriminative fear conditioning was carried out over five consecutive days and the procedure on each day was virtually identical. First, the mouse was connected to the recording equipment and placed in the 'neutral context', i.e. a chamber in which the mouse had been previously habituated for at least 2 hours and in which they never received shocks. The potential was then applied to the T_{O2} electrode for 10 minutes before the experiment began to ensure that T_{O2} signals were stable. Day 1, pre-exposure, was performed entirely in the neutral context: five tone (2900 Hz) and five white noise stimuli (both 30 s duration, 80 dB) were presented in pseudorandom order with a mean inter-stimulus interval of 80 s (range 60-100 s), with no shocks administered. On training days 1-3, the mouse was placed into the neutral context for 10 minutes and then transferred to one of the conditioning chambers (e.g. context A). Mice then received five tone and five white noise stimuli (the same as during pre-exposure), but now one of the

stimuli (counterbalanced across mice and across genotypes) was always paired with co-terminating footshock (0.3 mA, 0.5 s). On day 5, the fear memory recall test, mice were placed first into the neutral context for 10 minutes and then placed into a novel conditioning chamber (e.g. context B if trained in context A) and the five tone and five white noise stimuli were played with no shocks administered. Behavior (freezing) was monitored via a video camera in the roof of the chamber.

Acoustic Startle

To test for potential hearing impairments in 5-HTTOE mice, startle responses to acoustic stimuli of different intensities were measured in a separate cohort of mice (WT: n = 6; 5-HTTOE: n = 6) using the SR-Lab System (San Diego Instruments, San Diego, CA, USA). The test session began by placing a mouse in the Plexiglas cylinder where it was left undisturbed for 5 minutes. The test session consisted of eight 40 ms trial types at different sound intensities: 65, 70, 75, 80, 90, 100, 110, and 120 dB. Note that the 65 dB stimulus was identical to the background noise and was used to measure baseline movement in the cylinders. Five blocks of the eight trial types were presented in a pseudorandom order such that each trial type was presented once within a block of eight trials. The average inter-trial interval was 15 seconds (range: 10 to 20 seconds). The startle response was recorded for 65 ms (measuring the response every 1 ms), starting with the onset of the startle stimulus. The average startle amplitude recorded during the 65 ms sampling window was used as the measure of animals' reactivity to sounds. The data were analyzed using a general linear model with genotype as a between subjects factor, sound intensity as a within-subjects factor and body weight as a covariate.

Data Analysis

Behavior

Freezing during the discriminative fear conditioning paradigm was measured using a script in NIH Image (13), which compared consecutive video frames (1 Hz sampling) for pixel changes and assigned a freezing score if the % pixel change was below a set threshold calibrated for an absence of movement except for breathing (14). The freezing difference score was calculated as follows: % freezing during the 30 s cue presentation minus % freezing during the 30s before cue presentation (i.e. positive freezing scores indicate increased freezing to the cue and negative freezing scores indicate decreased freezing to the cue relative to the pre-cue period).

Tissue oxygen (T_{O2}) signals

T_{O2} signals were first down-sampled to 100 Hz. Cue-evoked T_{O2} responses were calculated by subtracting the mean T_{O2} signal in the 5 s before conditioned stimulus (CS) onset (i.e. baseline) from the T_{O2} signal during the 30 s CS presentation. This yielded a 30 s ΔT_{O2} signal, which was then divided into fifteen 2 s timebins (i.e. 0-2 s, 2-4 s, 4-6 s…28-30 s), with each data point equal to the mean value during each 2 s timebin (see Figure S2). Thus our T_{O2} data retain good temporal resolution (2 s), allowing us to analyze how the signals evolve during CS presentations, akin to event-related fMRI (15), rather than extracting a single response peak or area under the curve.

We have used an absolute ΔT_{O2} signal rather than a % change from baseline for the following reasons. First, there is occasionally considerable variation in the raw baseline signals (i.e. the background current) between animals. The precise reason for this variation is not known but could be due to differences in the active area of the electrode surface. Consider the raw T_{O2}

responses presented in Figures S2C and S2D. These were recorded from two different WT mice to a CS+ presentation at exactly the same point in training (training day 3, CS+4). The T_{O2} response in Figure S2C has a raw baseline signal of 605 nA whereas the T_{O2} response in Figure S2D has a raw baseline signal of 135 nA. Despite these baseline differences, the maximum signal changes evoked by the CS+ (i.e. the absolute ΔT_{O2}) are similar in both cases (+13 nA in Figure S2C, +11 nA in Figure S2D) whereas the % changes are very different (+2% in Figure S2C, +8% in Figure S2D). The second reason is that the raw T_{O2} signal can drift within a session, and this drift is not always linear. Under these circumstances, the same absolute signal change would yield different % signal changes for different trials within a single session. In short, subtracting a local baseline (e.g. the mean signal in the 5 s before CS onset), rather than using the % signal change from baseline, gives more reliable results in terms of the T_{O2} signal response.

During pre-exposure and training days, CS+ T_{O2} responses were averaged over the five CS+ trials and CS- T_{O2} responses were averaged over the five CS- trials of each session. On the fear memory recall day, we analyzed only the first CS+ versus the first CS- trial to mitigate the effects of extinction on subsequent presentations. For the correlational analysis with theta power, we calculated the mean CS+ evoked T_{O2} response (i.e. the mean value over the 15 time bins) for each mouse on each day of training and the fear memory recall day.

Local Field Potentials

LFPs were band-pass filtered between 1 Hz and 45 Hz. We calculated power spectra for the first 10 s of CS presentation. Spectra were then averaged over the five CS+ versus the five CS- trials for each mouse for each day. Spectra were computed in MATLAB (The Mathworks, Natick, MA, USA) using a fast Fourier transform size of 2000 samples with a Hamming window

(50% overlap) at a sampling rate of 1 kHz, and a frequency resolution of ~0.5 Hz. To compare across mice, the raw power spectra were normalized by expressing the power in each frequency bin ($\Phi_{i,}$) as a proportion of the total power between 1 and 45 Hz:

$$P_i = \left(\frac{\Phi_i}{\sum_1^{45} \Phi} \right)$$ where P_i = normalized power, Φ_i = raw power (mV^2)

An example of this normalization procedure is shown in Figure S2F-G. To compare theta power across genotypes, we calculated the sum of CS+ and CS- evoked theta power between 7-10 Hz for each mouse on each day. For the correlational analysis between T_{O2} response amplitude and theta power, we calculated a theta:delta ratio by dividing the sum of theta power between 7-10 Hz by the sum of delta power between 1-4 Hz. Spectrograms were generated in MATLAB, using a sliding time-window of 1 s, with 500 ms overlap, and a frequency resolution of ~0.25 Hz.

Histology

Determination of Electrode Placements

At the end of the fear conditioning experiment, mice implanted with recording electrodes were injected with sodium pentobarbitone; 200 mg⁄kg) and perfused transcardially with physiological saline (0.9% NaCl), followed by 10% formol saline (10% formalin in 0.9% NaCl). Their brains were removed and placed in 10% formol saline for 3 days, and then transferred to a 30% sucrose–formalin solution for 24 h and frozen. Coronal sections (40 µm) were then cut on a freezing microtome and stained with Cresyl violet to enable visualization of the recording sites. Only mice with confirmed electrode placements in the basolateral amygdala were used in the T_{O2} and LFP analyses (see Figure S1).

Quantification of Serotonin Transporter Binding Levels

Serotonin transporter binding levels in the amygdala were assessed in a separate group of mice (OE: *n* = 6; WT: *n* = 5; aged 3-6 months). Briefly, brains were snap frozen and sectioned, as described in Jennings *et al.* (1), and prepared for autoradiography using 2 nM [^{3}H]citalopram to determine serotonin transporter binding. Slides were exposed to [^{3}H]-sensitive Hyperfilm (Amersham Biosciences) and densitometric quantification of autoradiograms was performed, calibrated to tritiated tissue equivalents (Amersham Biosciences) and corrected for nonspecific signals. Optical densities from the basolateral amygdala of each hemisphere in three sections per animal were averaged and expressed as femtomoles per milligram of tissue (fmol/mg).

Neurochemical Analysis of 5-HT and 5-HIAA in Amygdala Tissue

High performance liquid chromatography with electrochemical detection was used to measure amygdala tissue levels of 5-HT. In a separate cohort of mice (*n* = 5 per group), tissue micropunches were obtained from the amygdala of WT and 5-HTTOE mice (16). Mice were euthanized by cervical dislocation and brains were rapidly removed, and frozen before being cut (150 µm sections) on a cryostat (-10°C). Amygdala tissue was micropunched (0.75 mm diameter) from 5 slices per mouse to give ~2.25 mg tissue/mouse/hemisphere. Samples were homogenized in 100 µl 0.06 M perchloric acid. Analytes were separated on Microsorb C18 reverse-phase columns (4.6 x 100-150 mm) and detected (glassy carbon working electrode set at +0.7 V versus Ag/AgCl; LC-4B electrochemical detector). The mobile phase consisted of 12.5% methanol, 0.13 M NaH_2PO_4, 0.85 mM EDTA and 0.01 mM sodium octane sulphonate at pH 3.55 (1 ml/min flow rate). Because of the low weight of tissue punches, data were expressed as picomoles/sample rather than picomoles/mg tissue.

Statistical Procedures

Behavioral analyses for the standard rodent fear conditioning paradigm were performed on 22 mice (WT: n = 11; OE: n = 11). Behavioral analyses for the discriminative fear conditioning paradigm were performed on 42 mice (WT: n = 22; OE: n = 20). T_{O2} analyses were performed on 33 mice (WT: n = 16; OE: n = 17), and LFP analyses were performed on 25 mice (WT: n = 12; OE: n = 13), all with histologically confirmed electrodes in the BLA and clean signals on all days of the experiment. Correlational analyses shown in Figure 5 were performed on 22 mice (WT: n = 10; OE: n = 12), with T_{O2} and LFP electrodes in the BLA and clean signals on all days of the experiment. Data were analyzed using *t*-tests, analysis-of-variance (ANOVA), or Pearson correlation in SPSS (version 15, SPSS Inc, IL, USA). ANOVAs are described using a modified version of Keppel's (17) notation in which the dependent variable is defined in the form: e.g. $A_2 \times B_3 \times S_{33}$, where A is a factor with two levels, B a factor with three levels, and S_{33} denotes that thirty-three subjects were included in the analysis. Interactions were investigated using simple main effects analyses with subsequent pairwise comparisons. The familywise error was set at $\alpha = 0.05$. Unless otherwise stated, all graphs show the mean ± 1 standard error of the mean (SEM).

Supplementary Results

Acoustic Startle

WT and 5-HTTOE mice exhibited indistinguishable acoustic startle responses to auditory cues ranging from 65 dB to 120 dB (no effect of genotype or genotype × sound intensity interaction, all $F < 2$, $p > 0.1$; see Figure S7), arguing against the possibility of any hearing impairments in 5-HTTOE mice.

Supplementary Discussion

Are Amygdala Theta Oscillations Locally Generated or Volume Conducted?

Several sources of evidence argue that theta oscillations are generated by amygdala neurons and do not simply reflect volume conduction from other brain regions. Pare and Collins have shown that single-unit activity is modulated by theta oscillations in the lateral amygdala of cats during discriminative fear conditioning (18). Intra-cellular recordings in guinea pigs and cats also confirm that theta oscillations can be induced in lateral amygdala neurons by near-threshold membrane depolarization via intracellular current injection (19, 20). Moreover, if amygdala theta was volume conducted, the most likely source would be the hippocampus as it exhibits high amplitude theta activity. In mice, Pape and colleagues have shown that theta activity in the BLA is synchronized with hippocampal theta during fear acquisition and recent retrieval of fear memories but not during fear extinction or remote fear retrieval or spatial exploration (21-24). Thus hippocampal-BLA theta-synchronization is not always present, which argues against passive volume conduction from the hippocampus. Also, hippocampal theta oscillations are strongly correlated with speed of movement (i.e. higher amplitude and higher frequency theta as running speed increases (25)), whereas theta oscillations in the BLA are strongest when mice are freezing (i.e. stationary). One interpretation of these datasets is that theta oscillations are intrinsically generated by amygdala neurons and are not volume conducted from the hippocampus (23).

However, against this, most models of neuronal oscillation assume that there must be spatial separation of current sinks and sources (in order to produce a dipole), and that these must be in coherently aligned neurons for oscillations to be observed at a population level in the LFP. In non-laminar structures, such as the amygdala, it is unclear whether the neuronal architecture

allows for the generation of theta oscillations at all. Resolution of this issue is not straightforward and would require multiple single unit and LFP recordings simultaneously from the amygdala and any candidate regions of theta generation; followed by silencing of those regions (e.g. by lesions) to see if amygdala theta oscillations persisted (for further discussion of this problem as it relates to the striatum, see Burke, 2005 (26)).

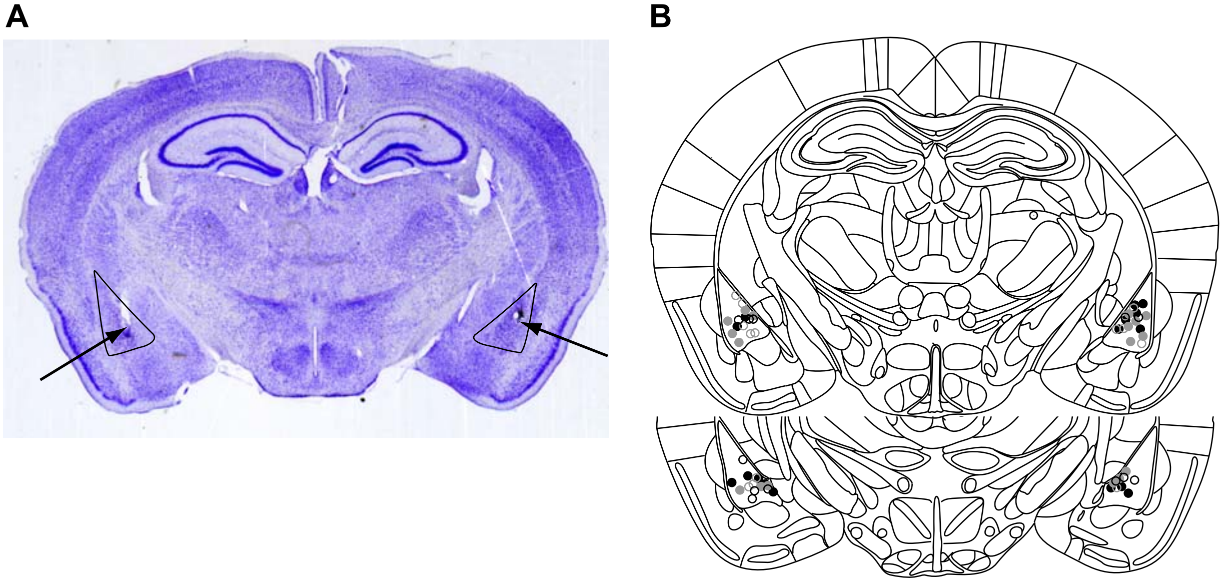

Figure S1. Histology. (**A**) Representative photomicrograph from one mouse with a carbon paste electrode (to measure T_{O2}) in the BLA of one hemisphere and a silver wire electrode (to measure LFPs) in the BLA of the other hemisphere. (**B**) Reconstructions of all BLA electrode positions in wild-type (black filled circles = CPEs; black open circles = LFP) and 5-HTT over-expressing mice (gray filled circles = CPEs; gray open circles = LFP). Figure adapted with permission from Paxinos & Franklin (2001) (27), Figures 45 and 47. BLA, basolateral amygdala; CPE, carbon paste electrode; LFP, local field potentials.

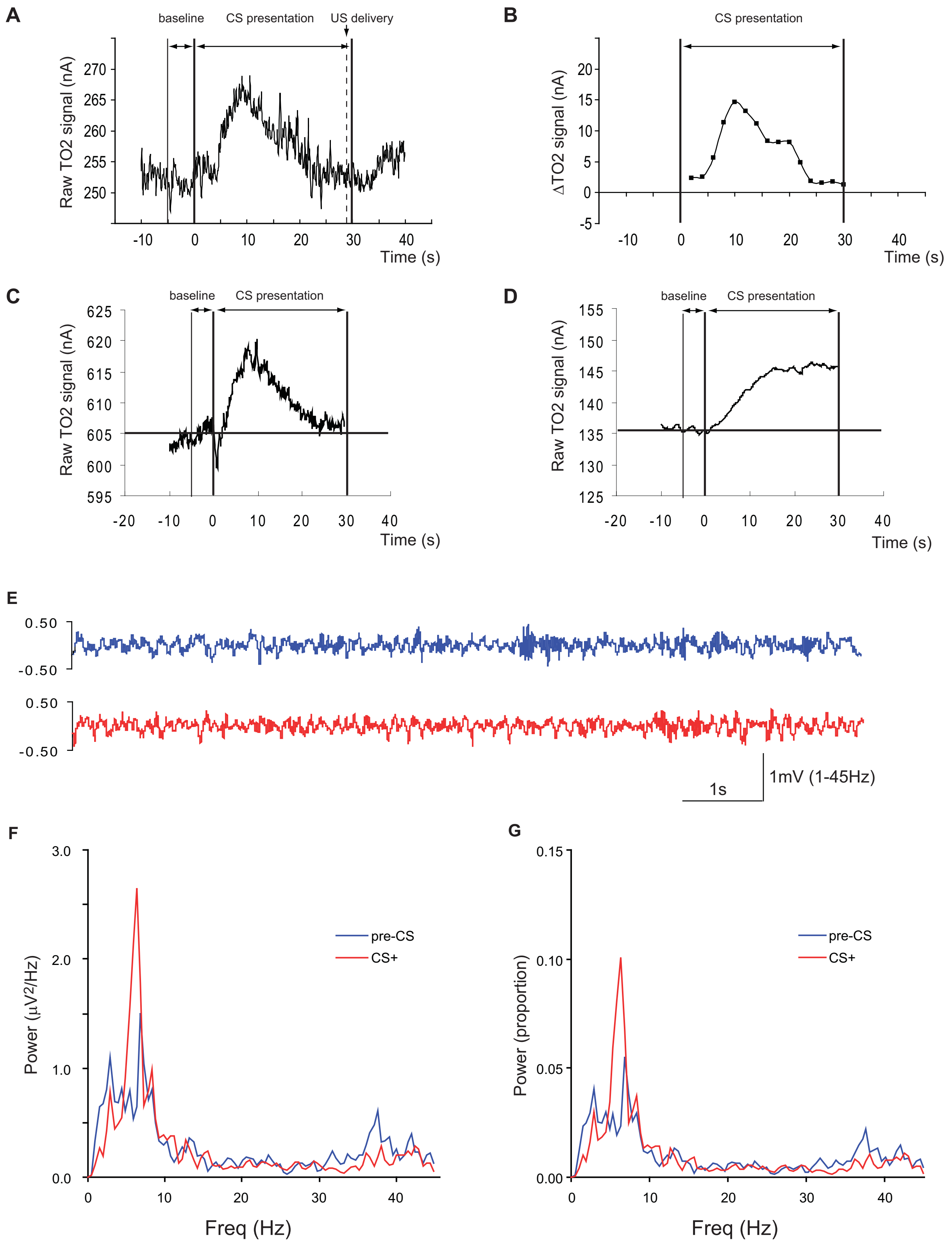

Figure S2. Methods for tissue oxygen (T_{O2}) and local field potential (LFP) analyses. (**A**) Raw T_{O2} data from the BLA during CS+ presentation (CS onset at 0 s, offset at 30 s). (**B**) Data

from (**A**) transformed into ΔT_{O2} signal by subtracting the baseline (mean T_{O2} signal during 5s before CS onset) and binning the mean T_{O2} signal into fifteen 2 s epochs. (**C-D**) Raw T_{O2} data illustrating the difference in the baseline signal between two WT mice. Despite these baseline differences, the magnitude of the CS+ evoked ΔT_{O2} response is similar in each mouse (max: +13nA in (**C**); +11 nA in (**D**)). These data demonstrate that subtracting a local baseline is the appropriate method for analysis, rather than using % signal change from baseline. (**E**) Bandpass-filtered (1-45 Hz) LFP recordings from the BLA in the 10 s periods before CS+ onset (blue) or after CS+ onset (red). (**F**) Data from (**E**) expressed as a power spectrum. In this example, CS+ onset evoked an increase in theta power compared to the pre-CS+ period. (**G**) Data from (**F**) normalized by expressing the power in each frequency bin as a proportion of the total power between 1-45 Hz. BLA, basolateral amygdala; CS, conditioned stimulus; US, unconditioned stimulus; WT, wild-type.

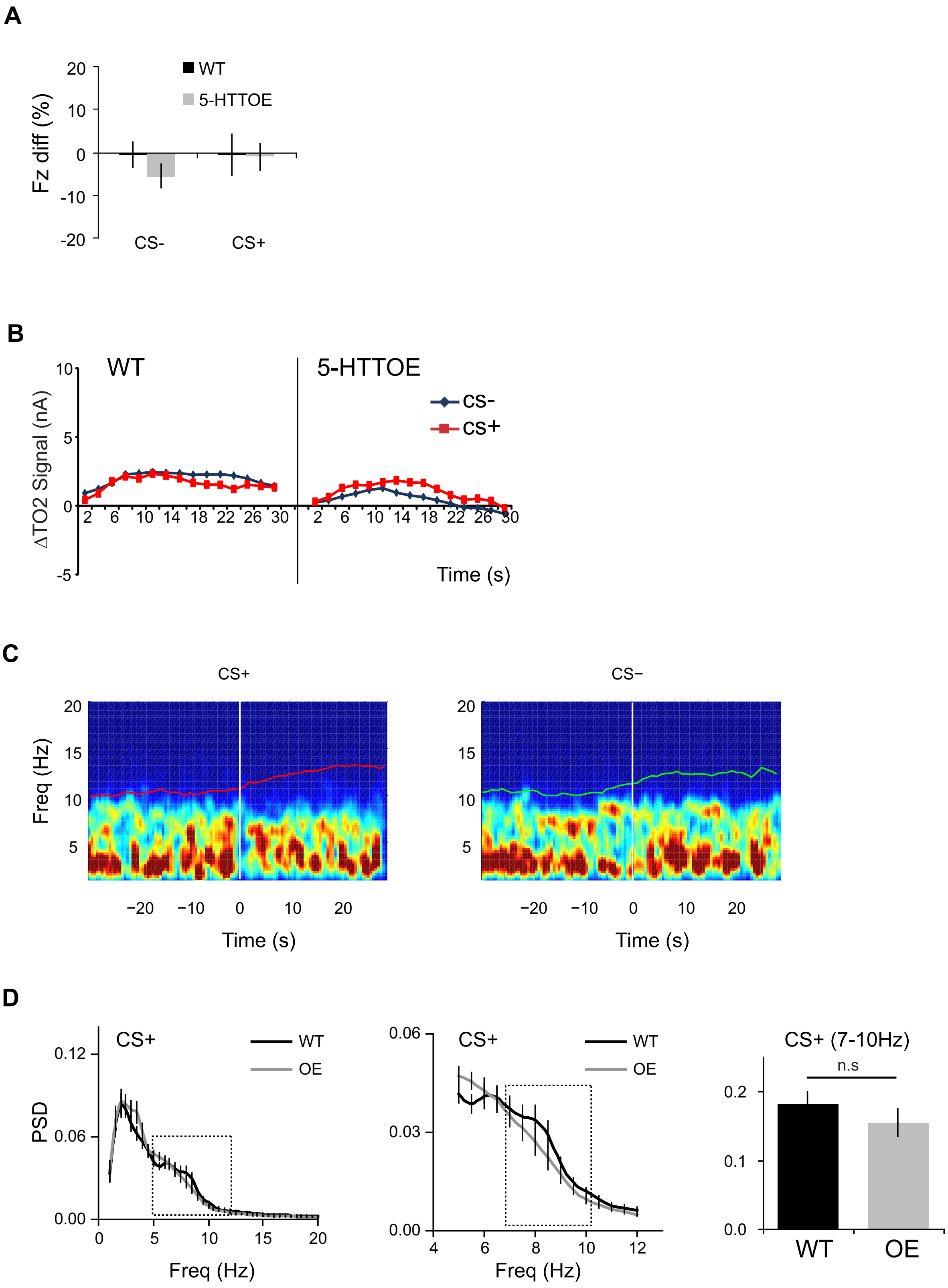

Figure S3. Discriminative fear conditioning: pre-exposure to the auditory cues. During the pre-exposure session, there were no differences between wild-type (WT) and 5-HTT over-

expressing mice (5-HTTOE) in terms of behavioral responses, tissue oxygen (T_{O2}) responses, or neuronal oscillations evoked by the auditory cues. (**A**) Freezing responses (% change in freezing compared to the baseline) to the 'to-be-allocated' CS+ and CS- stimuli (no effect of genotype, CS type, or interaction; all $F < 1$, $p > 0.4$). (**B**) T_{O2} responses (ΔT_{O2} change compared to the baseline) to the 'to-be-allocated' CS+ and CS- stimuli (no effect of genotype, CS type, or interaction; all $F < 2.1$, $p > 0.15$). (**C**) Example spectrograms from a WT mouse during 'to-be-allocated' CS+ (left) and CS- (right) trials. The 30 s before and after stimulus onset is shown, with stimulus onset at 0 as indicated by the white vertical line. The T_{O2} responses are superimposed in red (CS+) and green (CS-). Note, this is the same WT mouse as shown in the spectrogram in Figure 4A, which shows responses after fear conditioning. (**D**) Normalized power spectral density (PSD) plots for 'to-be-allocated' CS+ evoked responses in WT versus 5-HTTOE mice. The left panel shows spectra between 1-20 Hz; the middle panel shows spectra between 5-12 Hz (the boxed section in the left panel); the right panel shows the summed theta power between 7-10 Hz (the boxed section in the middle panel). There were no statistical differences between WT and 5-HTTOE mice ($F < 1.1$, $p > 0.3$). CS, conditioned stimulus.

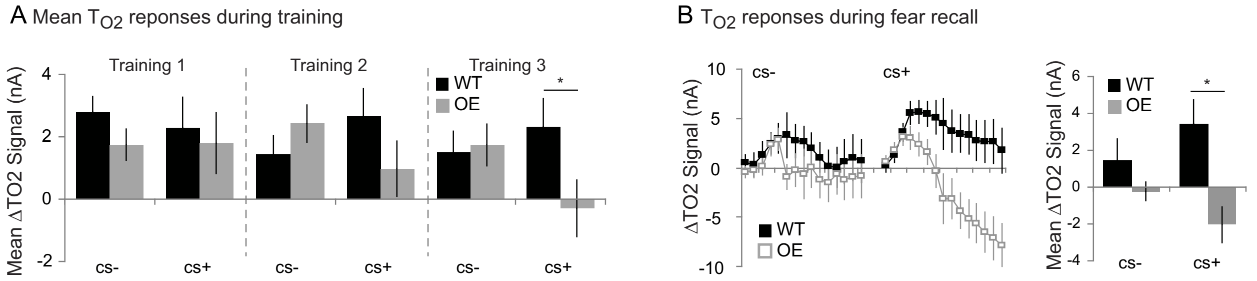

Figure S4. Aversive cue (CS+) and non aversive cue (CS-) evoked tissue oxygen (T_{O2}) signals in wild-type (WT) and 5-HTTOE (OE) mice during training and fear memory recall. (A) Mean ΔT_{O2} responses during the three training days. (B) ΔT_{O2} responses during fear memory recall.

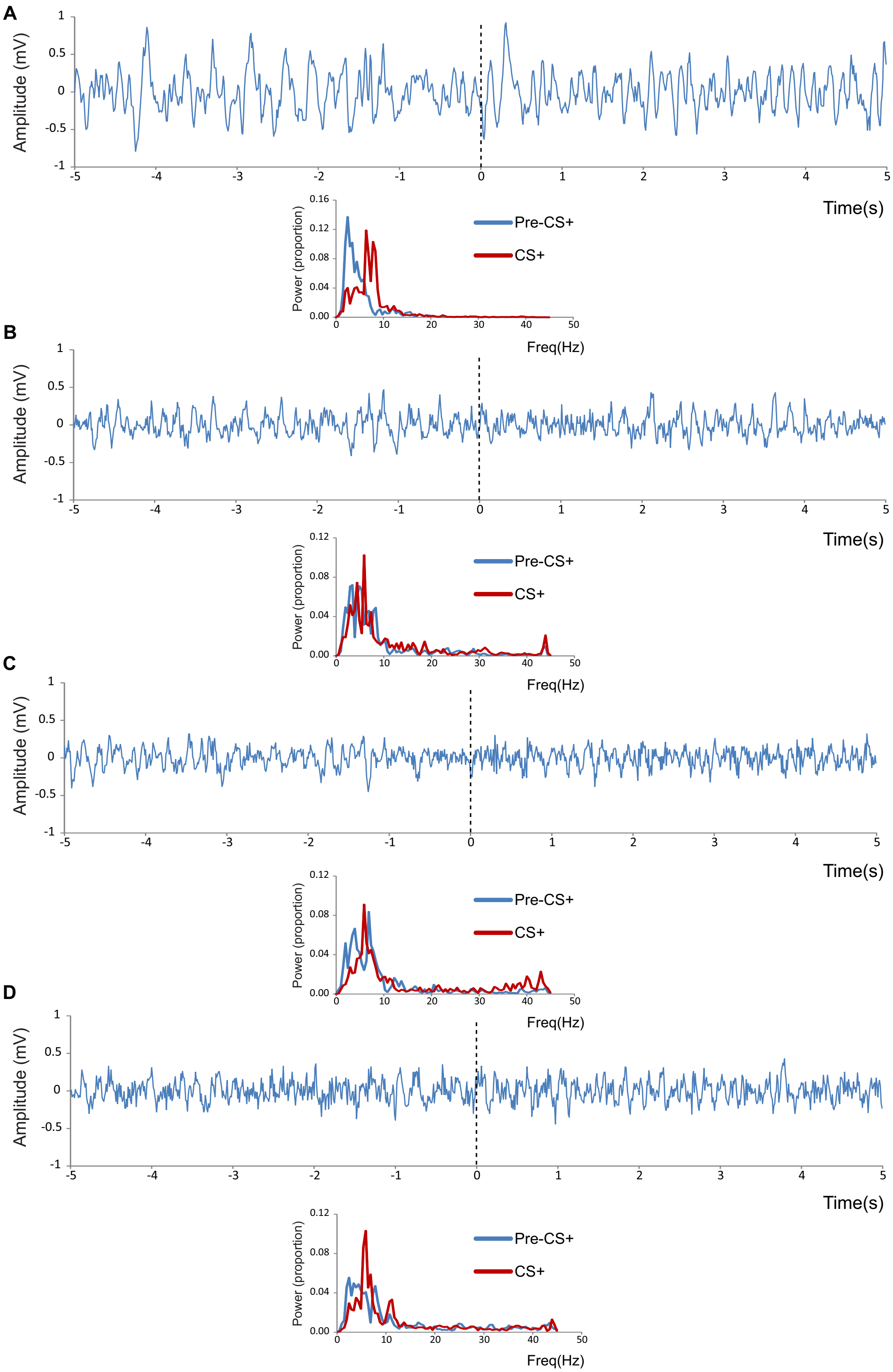
A
B
C
D
Amplitude (mV)
Time(s)
Power (proportion)
Freq(Hz)
Pre-CS+
CS+

Figure S5. Peri-stimulus responses to the CS+ on training day 3 in four different WT mice. (**A-D**) The top panel shows LFP amplitude (blue trace, in mV) for the 5 s before and 5 s after CS+ onset. The lower panel shows power spectra for the pre-CS+ period (blue) and the CS+ period (red) shown in the top panel. In each case, CS+ onset led to lower delta (1-4 Hz) power and higher peak theta power (5-10 Hz) compared to the pre-CS+ period. CS, conditioned stimulus; LFP, local field potential; WT, wild-type.

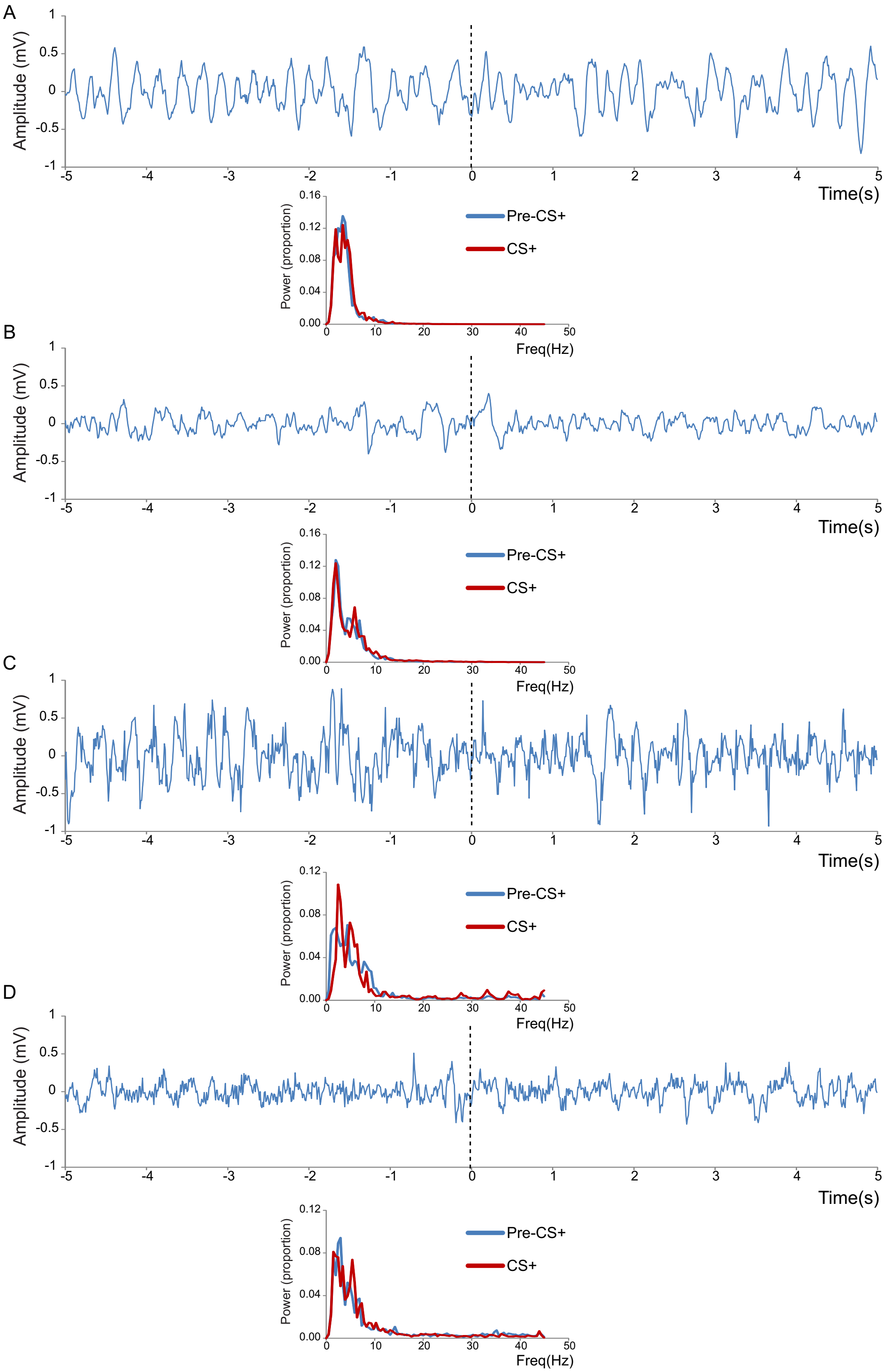
A
B
C
D
Amplitude (mV)
Time(s)
Power (proportion)
Freq(Hz)
Pre-CS+
CS+

Figure S6. Peri-stimulus responses to the CS+ on training day 3 in four different 5-HTTOE mice. (**A-D**) The top panel shows LFP amplitude (blue trace, in mV) for the 5 s before and 5 s after CS+ onset. The lower panel shows power spectra for the pre-CS+ period (blue) and the CS+ period (red) shown in the top panel. In **B-D** (but not A), CS+ onset led to slightly higher peak theta power (5-10 Hz) compared to the pre-CS+ period. However, the marked increases in theta power and the concomitant reductions in delta activity seen in WT mice were much less evident in 5-HTTOE mice. 5-HTTOE, serotonin transporter over-expressing; CS, conditioned stimulus; CSP, DEFINE; LFP, local field potential; WT, wild-type.

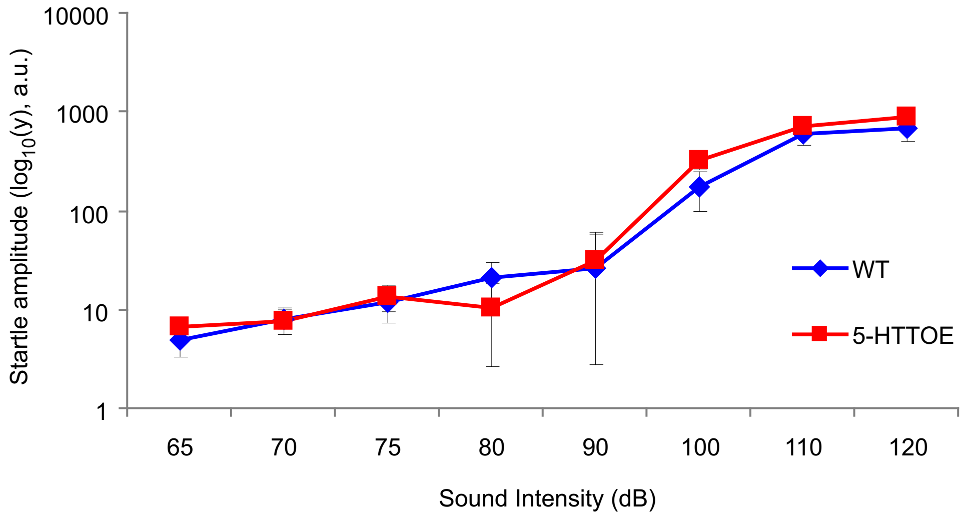

Figure S7. Acoustic startle responses. Wild-type (WT) and serotonin transporter over-expressing (5-HTTOE) mice exhibited indistinguishable acoustic startle responses to the auditory cues at all intensities (65-120 dB).

Table S1. 5-HTT expression levels and 5-HT tissue levels in the amygdala of wild-type and 5-HTT over-expressing mice.

	**WT**	**5-HTTOE**	***t*-test**
5-HTT	44.4 ± 5.5	121.4 ± 4.3	$t_{(9)} = 11.2$; $p < 0.001$
5-HT	7.6 ± 1.0	4.4 ± 0.9	$t_{(8)} = 2.5$; $p = 0.04$

Autoradiographically-determined 5-HTT binding and HPLC-determined 5-HT tissue levels in the amygdala of wild-type (WT) and 5-HTT over-expressing mice (5-HTTOE). Data are expressed as mean ± SEM fmol/mg for 5-HTT binding and pmol/tissue sample for 5-HT tissue levels.

Supplemental References

1. Jennings KA, Loder MK, Sheward WJ, Pei Q, Deacon RM, Benson MA, *et al.* (2006): Increased expression of the 5-HT transporter confers a low-anxiety phenotype linked to decreased 5-HT transmission. *J Neurosci*. 26:8955-8964.

2. O'Neill RD, Grunewald RA, Fillenz M, Albery WJ (1982): Linear sweep voltammetry with carbon paste electrodes in the rat striatum. *Neuroscience*. 7:1945-1954.

3. Rawlins JN, Feldon J, Gray JA (1979): Septo-hippocampal connections and the hippocampal theta rhythm. *Exp Brain Res*. 37:49-63.

4. Bolger FB, McHugh SB, Bennett R, Li J, Ishiwari K, Francois J, *et al.* (2011): Characterisation of carbon paste electrodes for real-time amperometric monitoring of brain tissue oxygen. *J Neurosci Methods*. 195:135-142.

5. McHugh SB, Fillenz M, Lowry JP, Rawlins JN, Bannerman DM (2011): Brain tissue oxygen amperometry in behaving rats demonstrates functional dissociation of dorsal and ventral hippocampus during spatial processing and anxiety. *Eur J Neurosci*. 33:322-337.

6. Hitchman ML (1978): *Measurement of Dissolved Oxygen*. New York: John Wiley.

7. Thompson JK, Peterson MR, Freeman RD (2003): Single-neuron activity and tissue oxygenation in the cerebral cortex. *Science*. 299:1070-1072.

8. Piilgaard H, Lauritzen M (2009): Persistent increase in oxygen consumption and impaired neurovascular coupling after spreading depression in rat neocortex. *J Cereb Blood Flow Metab*. 29:1517-1527.

9. Li J, Bravo DS, Upton AL, Gilmour G, Tricklebank MD, Fillenz M, *et al.* (2011): Close temporal coupling of neuronal activity and tissue oxygen responses in rodent whisker barrel cortex. *Eur J Neurosci*. 34:1983-1996.

10. McHugh SB, Marques-Smith A, Li J, Rawlins JN, Lowry J, Conway M, *et al.* (2013): Hemodynamic responses in amygdala and hippocampus distinguish between aversive and neutral cues during Pavlovian fear conditioning in behaving rats. *Eur J Neurosci*. 37:498-507.

11. Caesar K, Offenhauser N, Lauritzen M (2008): Gamma-aminobutyric acid modulates local brain oxygen consumption and blood flow in rat cerebellar cortex. *J Cereb Blood Flow Metab*. 28:906-915.

12. Offenhauser N, Thomsen K, Caesar K, Lauritzen M (2005): Activity-induced tissue oxygenation changes in rat cerebellar cortex: interplay of postsynaptic activation and blood flow. *J Physiol*. 565:279-294.

13. Schneider CA, Rasband WS, Eliceiri KW (2012): NIH Image to ImageJ: 25 years of image analysis. *Nat Methods*. 9:671-675.

14. Richmond MA, Murphy CA, Pouzet B, Schmid P, Rawlins JN, Feldon J (1998): A computer controlled analysis of freezing behaviour. *J Neurosci Methods*. 86:91-99.

15. Buchel C, Morris J, Dolan RJ, Friston KJ (1998): Brain systems mediating aversive conditioning: an event-related fMRI study. *Neuron*. 20:947-957.

16. Dalley JW, Chudasama Y, Theobald DE, Pettifer CL, Fletcher CM, Robbins TW (2002): Nucleus accumbens dopamine and discriminated approach learning: interactive effects of 6-hydroxydopamine lesions and systemic apomorphine administration. *Psychopharmacology (Berl)*. 161:425-433.

17. Keppel G (1982): *Design and analysis: a researcher's handbook*. 2nd ed. London: Prentice Hall.

18. Pare D, Collins DR (2000): Neuronal correlates of fear in the lateral amygdala: multiple extracellular recordings in conscious cats. *J Neurosci*. 20:2701-2710.

19. Pape HC, Pare D, Driesang RB (1998): Two types of intrinsic oscillations in neurons of the lateral and basolateral nuclei of the amygdala. *J Neurophysiol*. 79:205-216.

20. Pare D, Pape HC, Dong J (1995): Bursting and oscillating neurons of the cat basolateral amygdaloid complex in vivo: electrophysiological properties and morphological features. *J Neurophysiol*. 74:1179-1191.

21. Narayanan RT, Seidenbecher T, Kluge C, Bergado J, Stork O, Pape HC (2007): Dissociated theta phase synchronization in amygdalo- hippocampal circuits during various stages of fear memory. *Eur J Neurosci*. 25:1823-1831.

22. Seidenbecher T, Laxmi TR, Stork O, Pape HC (2003): Amygdalar and hippocampal theta rhythm synchronization during fear memory retrieval. *Science*. 301:846-850.

23. Pape HC, Pare D (2010): Plastic synaptic networks of the amygdala for the acquisition, expression, and extinction of conditioned fear. *Physiol Rev*. 90:419-463.

24. Lesting J, Narayanan RT, Kluge C, Sangha S, Seidenbecher T, Pape HC (2011): Patterns of coupled theta activity in amygdala-hippocampal-prefrontal cortical circuits during fear extinction. *PLoS One*. 6:e21714.

25. McFarland WL, Teitelbaum H, Hedges EK (1975): Relationship between hippocampal theta activity and running speed in the rat. *J Comp Physiol Psychol*. 88:324-328.

26. Burke JD (2005): Participation of striatal neurons in large-scale oscillatory networks In: Bolam JP, Ingham, C.A., Magill, P.J., editor. *The Basal Ganglia VIII*. New York: Springer, pp 25-35.

27. Paxinos G, Franklin KBJ (2001): *The Mouse Brain in Stereotaxic Coordinates*. 2nd ed. New York: Academic Press.
